# Roles of spinal V3 interneurons: Roles in controlling movement in healthy and injured conditions

**DOI:** 10.4103/NRR.NRR-D-25-00191

**Published:** 2025-09-03

**Authors:** Ruoying Zhang, Wei Wang, Xiaolong Zheng

**Affiliations:** 1 Department of Neurology, Tongji Hospital, Tongji Medical College, Huazhong University of Science and Technology, Wuhan, Hubei Province, China; 2 Hubei Key Laboratory of Neural Injury and Functional Reconstruction, Huazhong University of Science and Technology, Wuhan, Hubei Province, China; 3 Key Laboratory of Neurological Diseases of Chinese Ministry of Education, the School of Basic Medicine, Tongji Medical College, Huazhong University of Science and Technology, Wuhan, Hubei Province, China

**Keywords:** central nervous system, functional recovery, glutamatergic neuron, locomotion, neural circuit, nerve regeneration, sensorimotor, specific neuronal subpopulation, spinal cord injury, spinal V3 interneuron

## Abstract

Spinal V3 interneurons are glutamatergic neurons that are distributed among the dorsal, intermediate, and ventral spinal cord. They are involved in broad neural circuit connections in the central nervous system. Functionally, they play important roles in locomotion, such as the maintenance of robust and balanced gaits during walking. More importantly, after spinal cord injury, these neurons maintain their excitability and facilitate proprioceptive sensory transmission to motor neurons, which are crucial for the initiation of complex coordinated reciprocal and rhythmic activities that resemble locomotion. Thus, spinal V3 interneurons appear to be good candidates for the restoration of locomotion after spinal cord injury. Nevertheless, therapeutic strategies targeting spinal V3 interneurons for spinal cord injury are scarce. In this review, we summarize the functional roles of spinal V3 interneurons in locomotion across uninjured and injured states and come up with possible strategies targeting them to restore locomotor function after spinal cord injury. Currently, an increasing number of studies are dedicated to identifying spinal V3 interneurons and their roles in motor, sensory, and autonomic nervous system functions. However, there are still many unclear questions regarding the molecular and functional characteristics of V3 interneurons in the spinal cord, as well as their potential therapeutic effects after spinal cord injury. Future research should prioritize the in-depth characterization of this specific neuronal subpopulation based on its sensorimotor features to further enhance spinal cord repair and functional recovery.

## Introduction

Complex locomotor behaviors such as walking and swimming are generated by the functional operation of neural circuits, ultimately leading to the precisely coordinated activation of limb and body muscles (Kiehn, 2006, 2016; Arber and Costa, 2018). The planning and initiation of locomotion occur in supraspinal regions, including the motor cortex and brainstem (Lemon, 2008; Ferreira-Pinto et al., 2018; Arber and Costa, 2022; Leiras et al., 2022). However, rhythmic and patterned locomotion in mammals is primarily produced by the executive circuit that resides in the ventral spinal cord (Gosgnach et al., 2017). This spinal neural circuit is referred to as the central pattern generator (CPG), comprising diverse locomotor-related interneurons (Rybak et al., 2015; Haque and Gosgnach, 2019; Sengupta and Bagnall, 2023).

With the development of transgenic models, transneuronal techniques and single-cell technologies, vast populations of spinal neurons in CPG networks have been identified (Stepien et al., 2010; Shi et al., 2024; Zhang et al., 2024). Among these spinal neurons, locomotion is coordinated and regulated by dI6, V0–V3 and motor neurons (Sengupta and Bagnall, 2023). For example, V0 interneurons from the Dbx1 domain project predominantly commissural axons and are involved in left–right alternation (Lanuza et al., 2004; Talpalar et al., 2013; Bellardita and Kiehn, 2015). Ipsilaterally projecting V1 interneurons contribute to the regulation of locomotor frequency, as well as reciprocal flexor-extensor coordination (Gosgnach et al., 2006; Zhang et al., 2014; Kimura and Higashijima, 2019). V2 interneurons are responsible for hindlimb left–right alternation at high speeds (Crone et al., 2008, 2009).

**Figure 1:**
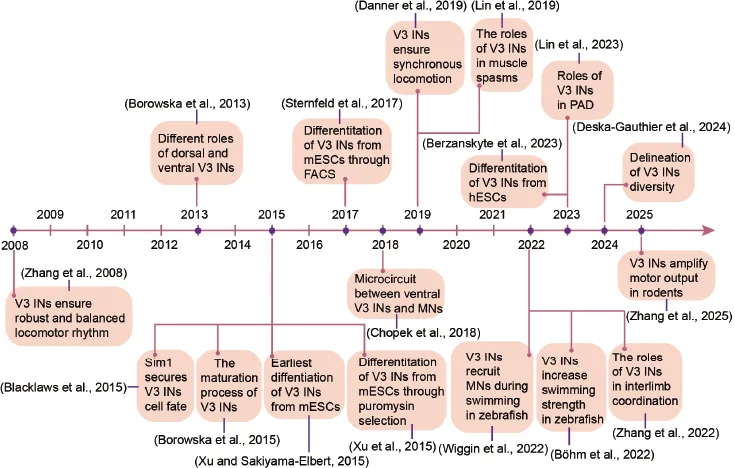
The timeline of the functional roles of spinal V3 INs in motor regulation. In this review, we examined key literature published over the past 20 years, covering the period from 2008 to 2025. Researchers have dedicated substantial time and effort over the years to elucidating the characteristics of V3 SpINs, thereby providing crucial evidence for their classification into subtypes, the neural circuits in which they participate, their functional roles in locomotion, and the differentiation methods used to enrich their populations. These key findings have created new opportunities for developing therapeutic strategies targeting V3 SpINs in spinal cord disorders. FACS: Fluorescence-activated cell sorting; hESCs: human embryonic stem cells; INs: interneurons; mESCs: mouse embryonic stem cells; MNs: motor neurons; PAD: primary afferent depolarization; SpINs: spinal interneurons.

Spinal V3 interneurons (V3 SpINs) from the most ventral Nkx2.2 progenitor domain are a group of excitatory commissural interneurons (Zhang et al., 2008). They contain distinct subpopulations with different laminar distributions and neuronal connectivity, contributing to a vast repertoire of locomotor functions (Deska-Gauthier et al., 2024). For example, they make monosynaptic connections with motor neurons and other locomotor-related interneurons in local neural circuits, thus depolarizing CPGs to increase locomotor output (Böhm et al., 2022; Wiggin et al., 2022; Zhang et al., 2025). In addition, they act as ascending long propriospinal neurons (aLPNs), connecting the cervical and lumbar spinal cord to control limb coordination during locomotion (Zhang et al., 2022). In addition, they receive extensive input from sensory afferents, serving as command neurons that ultimately amplify locomotor output (Laflamme et al., 2023; Lin et al., 2023). More importantly, after spinal cord injury (SCI), V3 SpINs facilitate sensory transmission to motor neurons and increase pattern generator activity (Lin et al., 2019, 2023; Bertels et al., 2022; Dougherty, 2023). In this way, they play important roles in initiating complex coordinated activity that resembles locomotion, which may be essential for the recovery of residual motor function after SCI (Lin et al., 2019). Considering the broad involvement of V3 SpINs in spinal neural circuit formation and sensorimotor functions, they could be potential therapeutic targets for SCI. Nonetheless, the relevance of targeting V3 SpINs in clinical practice has far lagged behind their role as key players in locomotion. Therefore, the focus of this review is to reflect on the current efforts toward understanding the heterogeneity of V3 SpINs, their ability to modulate normal motor functions and their contribution to neuroplasticity after SCI (**Figure 1**). Furthermore, we also offer a perspective on how they could be therapeutically targeted after SCI.

## Search Strategy

Studies cited in this narrative review published from 1999 to 2025 were searched in the Web of Science and PubMed databases using the following keywords: “V3 interneuron,” “spinal interneuron,” “spinal cord,” “Nkx2.2,” “Sim1,” “spinal cord injury” and “differentiation.” The combination of above search terms was also used to improve the specificity of literature search.

## Differences of Spinal V3 Interneurons from Other Ventral Spinal Interneurons in Motor Regulation

At all spinal segmental levels, the ventral spinal cord houses various ventral SpINs, which fall into at least four cardinal classes according to their genetically identified transcriptional factors, termed V0–V3 interneurons (Alvarez et al., 2005; Francius et al., 2013). These cardinal classes can be further divided into a repertoire of interneuron subclasses with distinct anatomical positions, physiological characteristics, and motor functions (Al-Mosawie et al., 2007; Talpalar et al., 2013; Griener et al., 2015; Bikoff et al., 2016; Hayashi et al., 2018; Deska-Gauthier et al., 2024). Besides, other spinal neurons that are located in the ventral spinal cord, including dI6 interneurons (Andersson et al., 2012; Griener et al., 2017; Haque et al., 2018; Perry et al., 2019), cerebrospinal fluid-contacting neurons (Gerstmann et al., 2022; Nakamura et al., 2023) and ventral spinocerebellar tract neurons (Chalif et al., 2022) are also critical contributors to motor regulation. However, the differences in V3 SpINs in neurotransmitter type, settling position, neural circuit connection, and motor function from other ventral SpINs endow them with notable roles in motor regulation.

In the dorsal–ventral axis of the spinal cord, the final laminar position of SpINs is determined by early temporal and spatial mechanisms (Hayashi et al., 2018). V3 SpINs are widely distributed along the dorsal-ventral axis. They are not only located in the most ventral spinal cord (laminae VIII), where commissural SpINs reside, but also in the deep dorsal horn in laminae IV and V, where proprioceptive sensory afferents enter, whereas most other ventral SpINs reside in more intermediate positions (laminae VI and VII; Gosgnach et al., 2017). In the rostral–caudal axis of spinal cord, unlike other ventral SpINs that exhibit changes in mediolateral and dorsoventral positioning along the rostral–caudal axis, V3 SpINs are homogeneously distributed between brachial, thoracic, and lumbar spinal cord, with the proportion of their subtypes slightly changed (Francius et al., 2013; Sengupta and Bagnall, 2023).

The specific laminar position of V3 SpINs enables them to be recruited in specific neural circuits related to motor regulation. In the local CPG circuit, there are many SpINs that have direct projections to motor neurons. Some of them have inhibitory effects on motor neurons to ensure interlimb alternation, such as Renshaw cells and Ia inhibitory interneurons (Britz et al., 2015; Moore et al., 2015). Other ventral SpINs, such as one subclass of V0 interneurons named V0c interneurons, modulate the motor output through local cholinergic projections. Since most V0c interneurons project ipsilateral and descending axons, the depolarization of motor neurons is limited (Zagoraiou et al., 2009). Even V2a SpINs, a group of excitatory SpINs, only exert influence on left-right alternation without affecting locomotor intensity (Crone et al., 2008). Remarkably, the excitatory commissural V3 SpINs provide glutamatergic inputs to both contralateral and ipsilateral motor neurons (Zhang et al., 2008; Chopek et al., 2018). They increase the excitability of motor neurons and depolarize local CPG circuits, finally amplifying the motor output (Zhang et al., 2025). Furthermore, V3 SpINs not only innervate motor neurons but also receive excitatory inputs from motor neurons, forming an excitatory feedback loop to further amplify motor output (Chopek et al., 2018). Although there are also other ventral SpINs that receive projections from motor neurons, such as Renshaw cells, Ia inhibitory interneurons and ventral spinocerebellar tract neurons, Renshaw cells and Ia inhibitory interneurons only provide inhibitory inputs to motor neurons (Britz et al., 2015; Moore et al., 2015), and ventral spinocerebellar tract neurons only affect locomotor rhythm instead of locomotor intensity (Chalif et al., 2022). In addition, V3 SpINs provide excitatory inputs to bilateral rhythmic centers in the CPG circuit, which contribute to the transition of gaits from walk and trot to synchronized motor patterns, such as gallop and bound (Bellardita and Kiehn, 2015; Danner et al., 2019). Thus, V3 SpINs provide dominant excitatory projections to adjacent motor neurons and contribute to increased motor output. Besides, V3 SpINs not only affect local neural circuits but also act as aLPNs to mediate interlimb coordination (Zhang et al., 2022). V0v-Dbx1 and V2-Shox interneurons are two neural subtypes that act as long descending propriospinal neurons, maintaining postural stability and interlimb coordination (Ruder et al., 2016). In contrast, aLPNs have different roles in ensuring left-right coordination in a context-dependent manner. The silencing of them only disrupts interlimb coordination during walking on a high-friction surface without affecting locomotor rhythm and intralimb coordination (Pocratsky et al., 2020). In accordance with this, V3 SpINs not only have neural connections between the lumbar and cervical spinal cord, but also ensure left–right coordination, especially in a context-dependent manner (Zhang et al., 2025). Thus, V3 SpINs have specific laminar positions, neural circuit connections, and motor functions compared with other SpINs and are robustly involved in motor regulation.

More importantly, after SCI, V3 SpINs play an indispensable role compared with other spinal interneurons due to their neurotransmitter characterization. While some excitatory spinal interneurons including V2a interneurons, undergo a reduction of excitatory neurotransmitters and acquire an inhibitory phenotype after SCI, V3 SpINs maintain their excitability, which may help increase the excitability of motor neurons and thus strengthen motor output (Bertels et al., 2022). Considering the specific and critical functional roles of V3 SpINs both in the intact and injured spinal cord, we provide comprehensive insights into this excitatory commissural interneuron and discuss below their indispensable roles in motor regulation.

## Developmental Process and Functional Diversification of Spinal V3 Interneurons

**Figure 2:**
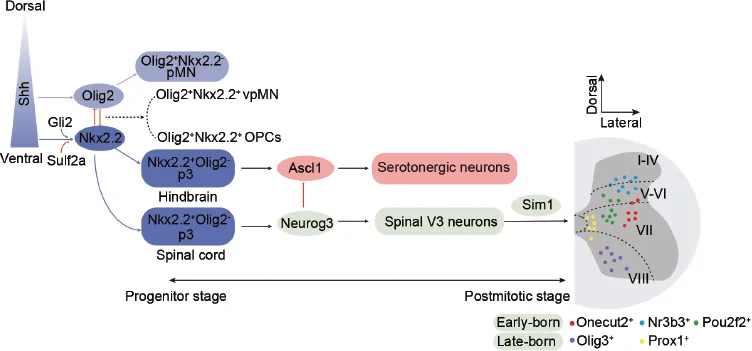
Developmental origins and specifications of spinal V3 interneurons. Spinal neurons are generated from neuronal progenitor domains in the neural tube. In the ventral neural tube, the patterning of progenitor cells depends on the graded activity of Shh. The dual interaction between Olig2 and Nkx2.2 in the ventral progenitor domains determines the fate of V3 SpINs, motor neurons and OPCs. Multiple transcription factors influence the generation of V3 SpINs. The Sulf2a protein controls Shh signaling by reducing Shh signaling levels, thus preventing their excess production. In contrast, the Gli2 protein responds to high Shh signaling and promotes their generation. Along the rostro-caudal axis, Neurog3 switches ventral progenitors from serotonergic to spinal V3 differentiation programs by repressing Ascl1 in p3 progenitors. After early V3 interneurons form, they undergo migration and specification. The cells characterized by the transcription factors Onecut^+^, Pou2f2^+^, and Nr3b3^+^ are early-born V3 SpINs that are distributed across the dorsoventral and mediolateral axes, whereas later-born V3 SpINs expressing Olig3^+^ and Prox1^+^ are restricted to more ventral and medial regions. As an important postmitotic marker of V3 SpINs, Sim1^+^ is indispensable for proper migration and commissural axonal guidance. OPCs: Oligodendrocyte precursor cells; Shh: Sonic hedgehog; SpINs: spinal interneurons; vpMN: ventral motor neuron progenitors.

V3 SpINs contain distinct subpopulations with different laminar distributions, physiological properties, and diverse neural circuit connectivity, which may underlie different locomotor functions (Deska-Gauthier et al., 2024). Their developmental process, with early temporal and spatial mechanisms, determines their heterogeneity (**Figure 2**).

### Establishment of the progenitor domain during early embryonic development contributes to the diversification of spinal V3 interneurons

During early embryogenesis, the graded activity of diffusible morphogens such as Sonic hedgehog (Shh), bone morphogenic protein, and Wnt signaling along the dorsal-ventral axis of the neural tube contributes to the formation of distinctive progenitor domains (Nordström et al., 2006; Alaynick et al., 2011). In the ventral neural tube, the generation of motor neurons and diverse interneurons depend on the graded activity of Shh (Briscoe et al., 2000; Jessell, 2000). The sequential activation of two transcription factors, Nkx2.2 and Olig2, in response to graded Shh signaling plays a primary role in ventral neural patterning to distinguish V3 SpINs from motor neurons and oligodendrocyte precursor cells (Briscoe et al., 1999; Gotoh et al., 2012; Jarrar et al., 2015; Jang et al., 2024; **Figure 2**). During patterning establishment, *Olig2* is first activated in response to Shh. With the increase of Shh activity, the high-threshold Shh-responsive gene *Nkx2.2* is sequentially activated, which in turn represses *Olig2*, thus leading to the formation of spinal V3 progenitor cells (Danesin et al., 2021; **Figure 2**). Mutation of *Nkx2.2* leads to the generation of motor neurons rather than V3 SpINs (Briscoe et al., 1999; Holz et al., 2010; Jarrar et al., 2015). Additionally, persistent expression of *Olig2* also inhibits the establishment of V3 SpINs. In the chick neural tube, the co-expression of Olig2 with Nkx2.2 leads to the generation of oligodendrocyte precursor cells (Sun et al., 2001). Besides, the co-expression of NKX2.2 and OLIG2 gives rise to ventral motor neuron progenitors during human embryonic neurogenesis, which represent a transient cell state that either downregulates NKX2.2 to adopt OLIG2^+^ classical motor neuron progenitor identity or downregulates OLIG2 and differentiates into NKX2.2^+^ V3 SpINs (Jang et al., 2024; **Figure 2**). Consequently, the dual interaction between NKX2.2 and OLIG2 determines the specification of V3 SpINs. Moreover, Shh mediates the interaction between Nkx2.2 and Olig2 through several mechanisms. A novel transcription factor named Sulf2a controls Shh-mediated cell type specification during spinal cord development. Sulf2a prevents the high-threshold Shh response and the activation of Nkx2.2, thus preventing the overproduction of V3 SpINs (Danesin et al., 2021; **Figure 2**). In addition to the Nkx2.2 and Olig2 proteins, Gli proteins are also transcription factors that function downstream of Shh signaling. In response to high levels of Shh, Gli2 plays the most important role in the generation of V3 SpINs, whereas Gli1 and Gli3 contribute to a lesser extent. Mutation of Gli2 and Gli3 results in the deficiency of V3 SpINs and defects in the segregation of other ventral neuronal progenitors (Bai et al., 2004; Lei et al., 2004; **Figure 2**).

The different neuronal cell types are also well-organized along the rostro-caudal axis. In the developing neural tube, hindbrain serotonergic neurons and V3 SpINs are both produced from ventral p3 progenitors and express the same transcription factors, Nkx2.2^+^, Foxa2^+^, and Ascl1^+^ (Jacob et al., 2013). However, how these two different cell types are distinguished from each other remains unknown. The transcription factor neurogenin3 (Neurog3) switches the ventral neuronal fate from serotonergic neurons to V3 SpINs by repressing Ascl1 in spinal p3 progenitors, thus promoting spinal V3 specification instead of serotonergic neurons in the spinal cord (Jacob et al., 2013; Carcagno et al., 2014; **Figure 2**).

Taken together, multiple identified factors and mechanisms facilitate the generation and differentiation of V3 SpINs (**Figure 2**).

### Timing of neurogenesis contributes to spinal V3 subpopulation specification during postmitotic time

After spatially and molecularly discrete progenitor domains are established during early embryonic development, mitotic progenitor cells complete their final division phase and exit the cell cycle, becoming postmitotic cells. Later, neurogenesis timing plays an essential role in ordering these postmitotic cells into their specific locations and promoting their physiological and functional diversification (Deska-Gauthier et al., 2020).

V3 SpINs are generated from the ventral-most p3 progenitor domain. As p3 progenitor cells exit their final cell cycle, they express the Sim1^+^ transcription factor and are defined as postmitotic V3 SpINs (**Figure 2**). These neurons then form divergent migratory streams and begin to express diverse transcription factor combinations (Deska-Gauthier et al., 2020). The timing of neurogenesis leads to the separation of postmitotic V3 SpINs into early-born and late-born V3 SpINs. Early-born V3 SpINs appear at embryonic day 9.5 (E9.5) to E10.5 and express the Onecut^+^, Pou2f2^+^, and Nr3b3^+^ transcription factors. They move laterally from the midline and are distributed across the dorsoventral and mediolateral axes in the spinal cord. Subsequently, late-born V3 interneurons expressing Olig3^+^ and Prox1^+^ transcription factors begin to migrate from E11.5–E12.5, and most of these V3 interneurons are restricted to more ventral and medial regions of the spinal cord (Deska-Gauthier et al., 2020, 2024; **Figure 2**). Different spinal V3 subtypes exhibit different axonal projections, physiological properties, as well as functional roles. For instance, early-born spinal V3 subtypes have predominantly ascending axon projections and act as aLPNs to mediate left–right alternation in walking and trot. However, late-born spinal V3 subtypes have both ascending and descending axonal projections, playing important roles in mediating left-right synchronization like gallop and bound (Deska-Gauthier et al., 2024). Notably, the transcription factor Sim1^+^ determines the diversity of V3 SpINs during neurogenesis (**Figure 2**). Although expressed in all V3 SpINs, it exclusively regulates the migration of dorsal and intermediate clusters. In the Sim1^+^ mutant group, the number of V3 SpINs in the dorsal laminae decreased, with a concomitant increase in the number of V3 SpINs in the intermediate laminae (Blacklaws et al., 2015; Deska-Gauthier et al., 2020).

After migration and laminar settling are completed, V3 SpINs are roughly segregated into dorsal (V3d) and ventral (V3v) subpopulations. They display distinct morphologies, axon projection profiles, and physiological properties. V3d SpINs have long ascending commissural projections and more complex morphology, whereas V3v SpINs have both ascending and descending projections with fewer processes. Moreover, these two V3 subtypes also exhibit distinct electrophysiological properties, neural circuit connections and functional divergence (Borowska et al., 2013). During maturation, the electrophysiological properties of these two spinal V3 subtypes are distinct (Borowska et al., 2015). In addition to being merely divided into dorsal and ventral subclasses, V3v SpINs can be further divided into smaller medial V3v SpINs and larger lateral V3v SpINs, which form focal layered microcircuits with motor neurons in the ventral spinal cord (Chopek et al., 2018).

The above-mentioned V3 SpINs are located in the lower thoracic and upper lumbar spinal cord, but how is their distribution in other parts along the rostro-caudal axis in the spinal cord? Immunofluorescence labeling combined with 3D imaging of the whole spinal cord revealed that the topographic distribution of V3 SpINs did not change significantly among the cervical, thoracic, or lumbar spinal cord, but the distribution of their subtypes slightly changed, with the number of V3d SpINs gradually increasing at the expense of V3v SpINs at the thoracic and lumbar levels (Francius et al., 2013).

In summary, the generation and patterning of V3 SpINs in the spinal cord are dependent on the synergistic contributions of various signaling pathways in the developing spinal cord, and their specification into diverse subtypes is attributed to early temporal and spatial mechanisms (**Figure 2**).

## Roles of Spinal V3 Interneurons in Spinal Neural Circuits

The heterogeneity of V3 SpINs, which is controlled by early spatial and temporal mechanisms, restricts their pre- and postsynaptic targets in spinal neural circuits. Determining the complex neural network in which V3 SpINs are involved is important for understanding their functional roles in the modulation of motor and sensory systems.

### Afferent inputs to spinal V3 interneurons

**Figure 3:**
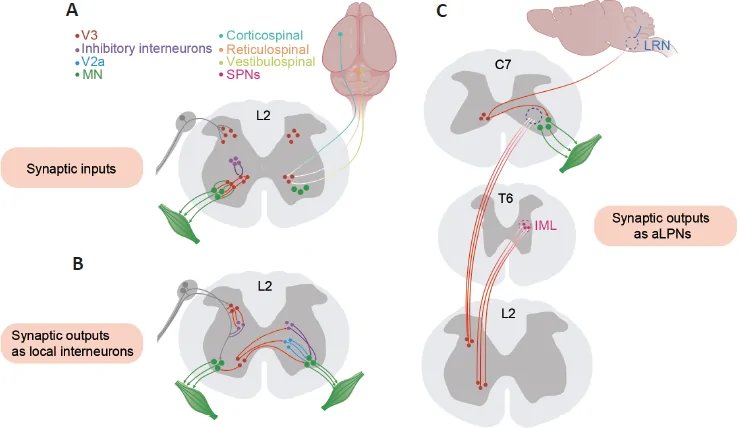
Integration of spinal V3 interneurons in spinal motor networks. (A) The afferent inputs that V3 SpINs receive. V3 SpINs form glutamatergic synapses with each other. They receive direct inhibitory input from other inhibitory interneurons and glutamatergic input from motor neurons. They also receive direct projections from primary sensory neurons (both proprioceptive group I afferents from muscles and low-threshold cutaneous afferents) located in the dorsal root ganglia and from corticospinal neurons, reticulospinal neurons, and vestibulospinal neurons in supraspinal regions. (B) Synaptic outputs of V3 SpINs in local CPG circuits. V3 SpINs project to neurons involved in locomotion. They provide glutamatergic drive to motor neurons from both the ipsilateral and contralateral sides of the spinal cord. Additionally, they form glutamatergic synapses with other locomotor-related interneurons, such as inhibitory interneurons and V2a interneurons. In addition, they directly innervate proprioceptive afferents (including Ia), which suggests their prominent roles in mediating sensory transmission in locomotion. (C) Synaptic outputs of V3 SpINs as long propriospinal neurons. Lumbar V3 SpINs provide glutamatergic synaptic output to thoracic SPNs, suggesting possible communication between the motor and autonomic systems. They also project cervical enlargements in the spinal cord, controlling coordination between the forelimbs and hindlimbs. V3 SpINs, as premotor interneurons in the cervical spinal cord, project to the contralateral LRN territory in the brainstem. This spinal-LRN-cerebellum-deep cerebellar nuclei loop is formed to update descending pathways in a timely fashion with ongoing activity from the spinal cord. aLPNs: Ascending long propriospinal neurons; CPG: central pattern generator; IML: intermediolateral column; LRN: lateral reticular nucleus; MN: motor neuron; SpINs: spinal interneurons; SPNs: sympathetic preganglionic neurons.

In local CPG neural circuits, V3 SpINs receive afferent inputs from diverse locomotor-related neurons. For instance, they are innervated by other V3 SpINs. As mentioned above, the ventral spinal V3 subclass can be divided into medial V3v SpINs and lateral V3v SpINs. Lateral V3v SpINs receive inputs from medial V3v SpINs, which in turn project to ipsilateral motor neurons (Chopek et al., 2018). The transmission between themselves integrates information from the sensorimotor system and ultimately activates motor pools. Moreover, lateral V3v SpINs also receive recurrent excitatory innervation from ipsilateral motor neurons (Chopek et al., 2018; **Figure 3**). This positive feedback loop may ensure motor stability. In addition, V3 SpINs also receive GABAergic innervation from inhibitory interneurons and cholinergic innervations from either motor neurons or V0c interneurons (Zhang et al., 2025), which may underlie the complex modulation of motor outputs (**Figure 3**).

In addition to participating in local neural circuits within the ventral spinal cord, V3 SpINs also receive abundant afferent inputs from the sensory system. The sensory feedback circuits transmit a wealth of sensory information from distinct parts of our body and limbs to spinal neurons in the spinal cord, thereby converging and integrating sensory information to modulate locomotor movements (Wang et al., 2022). The dorsal V3 SpINs distributed in the deep dorsal horn of the spinal cord receive extensive direct sensory afferent inputs comprising both proprioceptive (group I afferents from muscles) and cutaneous (low-threshold cutaneous afferents) sources, which control the pattern of locomotion (Lin et al., 2019, 2023; Laflamme et al., 2023; **Figure 3**). For example, they are involved in crossed reflex pathways, which transform sensory information from one limb to the contralateral limb. They can either directly stimulate contralateral motor neurons to initiate movement or indirectly suppress the contralateral motor response by connecting with other inhibitory interneurons. Thus, V3 SpINs are involved in both excitatory crossed reflexes and inhibitory crossed reflexes (Laflamme et al., 2023; **Figure 3**). In addition, V3 SpINs can modulate sensory transmission through another pathway. When a muscle is stretched, sensory feedback induces depolarization of sensory afferents across the spinal cord, a phenomenon known as primary afferent depolarization (PAD), which prepares the entire limb to respond to potential subsequent disturbances. V3 SpINs mediate this sensory-evoked PAD through a trisynaptic circuit. They receive extensive sensory innervation, including Ia afferent input, project across multiple spinal segments, and activate a population of GABAergic neurons that subsequently engage GABAA receptors on afferents to induce PAD (**Figure 3**), a process specifically referred to as GABA PAD (Lin et al., 2023). In summary, V3 SpINs receive multiple sensory afferent inputs as the main source of motor control.

In addition to sensory inputs, V3 SpINs also receive motor commands from the brain. Corticospinal tract projections are essential for voluntary and dexterous movements (Wang et al., 2017; Sinopoulou et al., 2022). Corticospinal neurons innervate numerous premotor interneurons, including Nkx2.2^+^ V3 SpINs, although the average number of synapses on V3 SpINs is relatively low (Ueno et al., 2018). Moreover, V3v SpINs also receive extensive descending reticulospinal and vestibulospinal inputs, which are features of commissural lamina VIII neurons (Bannatyne et al., 2003; Kasumacic et al., 2015; **Figure 3**).

Furthermore, the different spatial distributions of V3 SpINs may account for the differing projections they receive. V3d SpINs may be activated by extensive sensory afferents, whereas V3v SpINs may receive intensive innervations from descending supraspinal projections (Borowska et al., 2013). To be more specific, early-born Pou2f2^+^ V3 interneurons receive early-arrived sensory afferents, whereas late-born Prox1^+^ V3 interneurons receive only later-arrived descending supraspinal inputs (Deska-Gauthier et al., 2024).

### Synaptic outputs from spinal V3 interneurons

In addition to receiving synaptic inputs from multiple spinal and supraspinal regions, V3 SpINs also project various postsynaptic targets. For example, they directly connect to multiple locomotor-related neurons in CPG networks (**Figure 3**). Retrograde transsynaptic labeling demonstrated that these neurons make direct synaptic connections with contralateral motor neurons (Zhang et al., 2008). In addition, V3 SpINs have been shown to form focal layered microcircuits with ipsilateral motor neurons via electrophysiology and holographic photostimulation (Chopek et al., 2018). In addition, they contact other locomotor-related interneurons, such as Renshaw cells, V2 cells, and other unidentified inhibitory interneurons (Zhang et al., 2008; **Figure 3**). Notably, V3 SpINs form long commissural axonal projections that travel across several spinal segments. Thus, the activation of V3 SpINs can lead to rapid depolarization of CPG networks at all lumbar segments to amplify motor outputs (Lin et al., 2023). Multiple computational models have been established to better delineate the participation of V3 SpINs in CPG networks. These excitatory interneurons provide mutual excitation between the left and right extensor rhythmic generator centers (Danner et al., 2016, 2019).

In addition to forming local excitatory neural circuits in the CPG network, a subset of V3 SpINs in the intermediate spinal cord (Nr3b3^+^ and Pou2f2^+^ subtypes) are also considered aLPNs, which reside in the lumbar spinal cord and have direct excitatory projections to the cervical spinal cord on the contralateral side (Zhang et al., 2022; Deska-Gauthier et al., 2024; **Figure 3**). In addition, V3 SpINs located in the lumbar region also provide synaptic input to sympathetic preganglionic neurons in the thoracic intermediolateral column region (**Figure 3**). By optically activating either the lumbar V3 interneurons in spinal cord preparations or their terminals in thoracic slice preparations, prolonged depolarizations or action potentials are recorded in sympathetic preganglionic neurons via whole-cell patch clamp. This work demonstrated that V3 SpINs mediate a direct intraspinal connection between the lumbar locomotor circuitry and the thoracic sympathetic network (Chacon et al., 2023).

In addition to innervating propriospinal neurons, V3 SpINs can also provide synaptic outputs to supraspinal systems. For example, they provide direct excitatory innervation to proprioceptive sensory afferents (including Ia), causing a glutamate receptor-mediated PAD (glutamate PAD), which contributes to the facilitation of sensory transmission to motor neurons, especially after SCI (Lin et al., 2023; **Figure 3**). In addition, they also act as spinal dual-axon neurons, with coincident inputs to forelimb motor neurons and the lateral reticular nucleus in the brainstem (Pivetta et al., 2014; **Figure 3**).

In summary, V3 SpINs are integrated into various supraspinal, propriospinal, sensory, and local interneuron pathways (**Figure 3**).

## Physiological Functions of Spinal V3 Interneurons

The extensive integration of V3 SpINs in spinal neural circuits endows them with functional roles as spinal cord command neurons that regulate motor outputs in the intact spinal cord.

### Roles of spinal V3 interneurons in motor outputs

**Figure 4:**
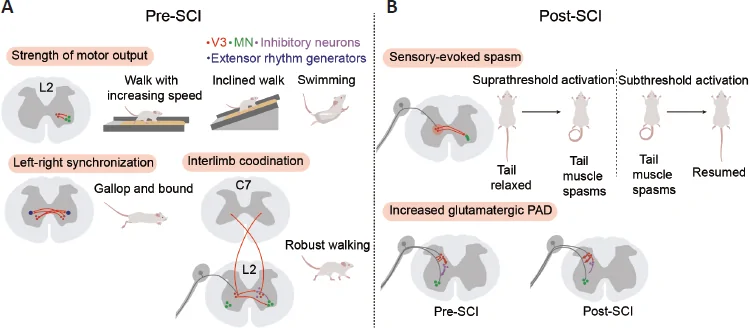
Functional roles of spinal V3 interneurons in locomotion. (A) Locomotor functions of V3 SpINs in the uninjured spinal cord. V3 SpINs directly synapse with motor neurons, which increases the strength of motor output in challenged locomotor tasks such as walking with increasing speed on the treadmill, walking on the inclined treadmill and swimming. They provide mutual excitation between bilateral extensor rhythm generators, which promotes left-right synchronization, ensuring animals to gallop and bound. The V3 SpINs in the lumbar spinal cord act as aLPNs that project cervical enlargement and are responsible for speed-dependent coordination. They also mediate excitatory and inhibitory crossed reflexes, transmitting somatosensory stimulation to the contralateral limb, which is important for robust and balanced walking. (B) The neuroplasticity of V3 SpINs during locomotion after SCI. V3 SpINs receive abundant inputs from primary sensory afferents, which mediate sensory-evoked muscle spasms after SCI. When given strong suprathreshold activation, V3 SpINs directly evoke muscle spasms. However, when given continuous subthreshold activation, sensory-evoked spasms are inhibited. V3 SpINs receive sensory inputs from both cutaneous and proprioceptive resources and innervate GABAergic interneurons, which causes GABA-mediated PAD. They also directly innervate proprioceptive afferents, facilitating glutamate-mediated PAD. After SCI, the glutamate-mediated PAD increases markedly, whereas the GABA-mediated PAD decreases or remains unchanged or decreased, probably facilitating sensory transmission to motor neurons. aLPNs: Ascending long propriospinal neurons; MN: motor neuron; PAD: primary afferent depolarization; SCI: spinal cord injury; SpINs: spinal interneurons.

**Figure 5:**
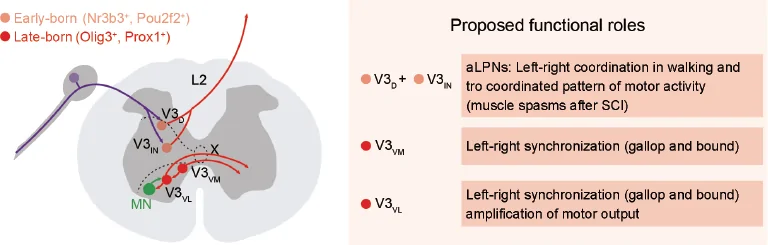
The association among birthdate, neural circuit connections and functional roles of spinal V3 interneurons. V3 SpINs are subdivided into early-born and late-born interneurons according to temporal and spatial mechanisms indicated in Figure 2. Early-born spinal V3 subtypes (Nr3b3^+^ and Pou2f2^+^) comprise V3D and V3IN, and they project predominantly ascending axons to rostral spinal segments, whereas late-born spinal V3 subtypes (Olig3^+^ and Prox1^+^) contains V3VM and V3VL, and they project both ascending and descending axons. Early-born spinal V3 subtypes act as aLPNs and contribute to left-right alternation in walking and trot, whereas late-born spinal V3 subtypes act as local excitatory commissural interneurons that play roles in left-right synchronization and motor output amplification. aLPNs: Ascending long propriospinal neurons; MN: motor neuron; SCI: spinal cord injury; SpINs: spinal interneurons; V3D: dorsal spinal V3 interneurons; V3IN: intermediate spinal V3 interneurons; V3VL: ventral lateral spinal V3 interneurons; V3VM: ventral medial spinal V3 interneurons.

Since V3 SpINs directly connect to motor neurons and locomotor-related interneurons both ipsi- and contralaterally, they mediate the strength of motor output to adapt to changes in internal or external forces. The optogenetic activation of V3 SpINs in the zebrafish spinal cord leads to an increase in the probability of starting a swim bout, the duration of the swim bouts, and the amplitude of the tail oscillations during swimming. In contrast, ablation of them via diphtheria toxin-A decreases swimming speed (Böhm et al., 2022). Similarly, after laser ablation of V3 SpINs during *in vivo* fictive locomotion in zebrafish, the proportion of active motor neurons during swimming was reduced without affecting locomotor frequency (Wiggin et al., 2022). Thus, V3 SpINs participate in regulating locomotor amplitude by recruiting spinal motor neurons during spontaneous fictive locomotion (**Figure 4** and **Additional Table 1**). Moreover, a previous study has revealed the role of V3 SpINs in depolarizing and amplifying motor neuron output both *in vitro* and in adult mice. The optogenetic activation of V3 SpINs in neonatal spinal cord preparations broadly initiates bilateral motor neuron excitation throughout the lumbar spinal cord, especially in the extensor motor pool (Zhang et al., 2025). Consistent with *in vitro* evidence, the localized optogenetic activation of V3 SpINs in awake adult mice also evokes widespread motoneuron excitation in multiple flexor and extensor muscles, with muscle activity in extensor muscles being greater than that in flexor muscles (Zhang et al., 2025). Furthermore, the silencing of V3 SpINs by blocking glutamate transmission leads to the limited walking speed of mice on the treadmill (Zhang et al., 2022), as well as the failure of demanding tasks that require increased extensor muscle strength (Zhang et al., 2025). In conclusion, V3 SpINs play a critical role in amplifying motor outputs, especially for extensor motor pools, either by helping produce weight support during standing and walking or increasing muscle force during demanding locomotor states (Zhang et al., 2008, 2022, 2025; Borowska et al., 2013; Chopek et al., 2018; Danner et al., 2019; Lin et al., 2019, 2023; Böhm et al., 2022; Wiggin et al., 2022; Chacon et al., 2023; Laflamme et al., 2023; **Figures 4**, **5**, and **Additional Table 1**).

**Additional Table 1:** Roles of spinal V3 interneurons in neural circuits and motor function

Animal	Spinal cord model	Neural circuitry	Functional role	Reference
Zebrafish	Intact spinal cord	Innervation of motor neurons	Regulation of locomotion amplitude by recruitment of motor neurons	Wiggin et al., 2022
			Increase in swim strength by recruitment of motor neurons	Bohm et al., 2022
Mouse	Intact spinal cord	Microcircuits between medial V3v, lateral V3v and motor neurons	Increase in motor outputs	Chopek et al., 2018
		Innervation of V2a interneurons and motor neurons	Increase in locomotor strength and speed	Zhang et al., 2025
		V3v: Innervation of motor neurons	V3v: Recruitment in both running and walking	Borowska et al., 2013
		V3d: Innervation by sensory inputs	V3d: Recruitment only in running	
		Innervation of motor neurons, V2 interneurons and inhibitory interneurons	Maintenance of robust and balanced locomotion	Zhang et al., 2008
		Innervation of the left and right extensor rhythm generators	Promotion of left-right synchronized locomotion	Danner et al., 2019
		Innervation of cervical enlargement by lumbar V3 interneurons	Maintenance of high-speed locomotion and interlimb coordination	Zhang et al., 2022
		Innervation of thoracic SPNs by lumbar V3 interneurons	Communication between motor and autonomic systems	Chacon et al., 2023
		Innervation by sensory inputs and projection to contralateral motor neurons	Maintenance of interlimb coordination and stability during locomotion	Lin et al., 2023
	Sacral spinal cord transection	Innervation by sensory inputs	Initiation of complex coordinated reciprocal and rhythmic activity (muscle spasms)	Lin et al., 2019
		Innervation by sensory inputs and projection to inhibitory interneurons/Innervation of sensory inputs	GABA PAD/glutamate PAD	Laflamme et al., 2023

GABA: Gamma-aminobutyric acid; PAD: primary afferent depolarization; V3d: dorsal spinal V3 interneurons; V3v: ventral spinal V3 interneurons.

Considering the heterogeneity of V3 SpINs, different V3 subtypes control different motor output patterns during task-dependent locomotion processes (**Additional Table 2**). For example, V3v SpINs are recruited during both running and swimming due to their role as premotor neurons that directly control motor outputs, whereas V3d SpINs serve primarily for running rather than swimming because of their dependence on sensory feedback from primary afferents (Borowska et al., 2013). To be more specific, two spatially overlapping but temporally separate Pou2f2^+^ and Prox1^+^ V3 interneurons also display different recruitment patterns. Early-born Pou2f2^+^ V3 interneurons receive sensory afferents at early embryonic time points and are widely recruited across various sensorimotor tasks, whereas late-born Prox1^+^ V3 interneurons lack sensory inputs and receive only descending supraspinal inputs, restricting their functional recruitment to only specific locomotor tasks, such as hindlimb loading and balance-dependent behaviors, but not swimming or speed-dependent walking on the treadmill (Deska-Gauthier et al., 2024).

**Additional Table 2:** Functional roles of spinal V3 interneuron subpopulation

V3 interneuron subtype	Neurogenesis timing	Laminar settling position	Axon projection	Functional recruitment
Onecut2+ V3	Early-born (E9.5-E10.5)	Intermediate positions within laminae VI, VII, and X	Descending ipsilateral axons	Unknown
Nr3b3+ V3 (V3d, aLPNs)	Early-born (E9.5-E10.5)	Predominantly laminae V	Ascending commissural axons (from lumbar to cervical spinal cord)	All overground locomotion (but not swimming)/ forelimb-hindlimb coordination
Pou2f2+ V3 (aLPNs)	Early-born (E9.5-E10.5)	Intermediate positions within laminae VI, VII, and X	Descending ipsilateral, descending commissural, and ascending commissural axons (from lumbar to cervical spinal cord)	All sensorimotor tasks (low speed stepping, intermediate speed stepping, inclined stepping, staggered stepping, lateral motion, and swimming)/ forelimb-hindlimb coordination
Olig3^+^ V3 (V3v)	Late-born (E11.5-E12.5)	Within ventral laminae (laminae VIII)	Descending commissural and ascending commissural	All overground locomotion, including swimming
Prox1^+^ V3	Late-born (E11.5-E12.5)	Intermediate positions within laminae VI, VII, and X	Local commissural axons	Balance- and weight-load-specific tasks (inclined stepping, staggered stepping, lateral motion)

E11.5-E12.5: Embryonic day 11.5 to embryonic day 12.5; E9.5-E10.5: embryonic day 9.5 to embryonic day 10.5; V3d: dorsal spinal V3 interneurons; V3v: ventral spinal V3 interneurons.

### Roles of spinal V3 interneurons in locomotor pattern generation

Rhythm generation and locomotor pattern regulation are the other essential parts of motor control and are highly dependent on local CPG circuits (Deska-Gauthier and Zhang, 2019). While V3 SpINs are not necessary for rhythm generation, they play crucial roles in locomotor pattern regulation. When synaptic transmission of V3 SpINs was attenuated by selective expression of the tetanus toxin light chain subunit *in vitro*, recordings of ventral roots revealed increased locomotor rhythm variance and greater variability in the step cycle period (Zhang et al., 2008). Similarly, when the excitability of V3 SpINs was acutely reduced by activation of the allatostatin receptor system, recordings of the ventral roots also revealed the same results. Furthermore, when allatostatin was applied to the lumbar spinal cord of awake adult mice, the mice presented abnormal gaits with meandering trajectories due to variability in the timing of both the stance and swing phases during walking (Zhang et al., 2008). Overall, these findings indicate that V3 SpINs are required for the production of a regular walking rhythm (**Figure 4** and **Additional Table 1**).

Additionally, the commissural V3 SpINs also mediate interactions between rhythm-generating locomotor circuits located on each side of the spinal cord and are necessary for left-right synchronization during locomotion (**Figures 4**, **5**, and **Additional Table 1**). By bilateral photoactivation, an increase in the duration and amplitude of integral electroneurogram bursts on the extensor side was recorded, suggesting that they activate bilateral extensor circuits. When light stimulation was shifted toward one side of the spinal cord, the duration of extensor bursts still increased on both sides but was more pronounced on the contralateral side than on the ipsilateral side. Thus, V3 SpINs mediate mutual excitation between the extensor centers of the left and right rhythm generators, which may support synchronized left-right activity, such as the gallop and bound during high-speed locomotion (Danner et al., 2019). In contrast, the ablation of V3 SpINs causes the loss of the synchronous gait (gallop and bound), with only walk and trot occurring (Danner et al., 2016), which supports their indispensable roles in the transition of gaits to gallop and bound in a high-speed context.

In addition to mediating mutual excitation between the left and right rhythm generators, a subpopulation of V3 SpINs also contributes to forelimb-hindlimb coordination (**Figures 4**, **5**, and **Additional Table 1**). Retrograde tracing revealed that the cell bodies of V3 SpINs in the lumbar spinal cord act as aLPNs that have direct excitatory projections to the contralateral cervical enlargement. When glutamatergic transmission was conditionally knocked out (V3OFF mice) in transgenic mice, the mice exhibited a lateral-sequence walking gait instead of a stable trot gait at low speeds and high variability in left-right coordination at high speeds (Zhang et al., 2022), which is consistent with the previous conclusion that V3 SpINs are essential for robust and balanced gait (Zhang et al., 2008). These findings suggest that spinal V3 aLPNs are critical for the control of interlimb coordination, whereas local spinal V3 CINs contribute to synchronized locomotion (**Figures 4**, **5**, and **Additional Table 1**).

Additionally, V3 SpINs maintain left-right coordination by connecting with inhibitory interneurons in CPG networks. That is, V3 SpINs transmit sensory signals to the contralateral side of the spinal cord and synapse with local inhibitory interneurons that indirectly exert an inhibitory influence on motor neurons. This inhibitory crossed reflex is also necessary for left–right coordination during locomotion (Laflamme et al., 2023).

As a result, V3 SpINs play important roles in regulating motor outputs and coordinating complex movements in the intact spinal cord. Different subsets of V3 SpINs selectively participate in context-dependent locomotion, adapting to changing requirements in the environment. That is, they are more likely to act as locomotion modulators, determining the right forms of motor action to take place (**Figures 4**, **5**, and **Additional Table 1**).

## Functional Roles of Spinal V3 Interneurons after Spinal Cord Injury

Traumatic SCI is caused by sudden insults, resulting in severe deficits in motor, sensory, and autonomic functions (Ahuja et al., 2017; GBD Spinal Cord Injuries Collaborators, 2023). SpIN networks would experience reorganization, contributing to both adaptive and maladaptive neuroplastic changes after traumatic SCI (Beauparlant et al., 2013; Punjani et al., 2023). As highlighted previously, V3 SpINs play diverse roles in shaping sensory and motor functions in the intact spinal cord. Understanding their post-injury functions is important for understanding their contribution to neuroplastic potential after SCI and how we can harness their neuroplasticity therapeutically.

In the chronic sacral SCI mouse model, V3 SpINs could initiate and maintain sensory-evoked tail spasms (**Figures 4**, **5** and **Additional Table 1**). When V3 SpINs are optogenetically activated, a complex motor pattern similar to spasms is recorded, and it is nearly the same as that of brief sensory stimulation. Moreover, the timing of sensory-evoked spasms can be reset by their activation (Lin et al., 2019). Given that V3 SpINs receive abundant sensory inputs, the sensory-evoked muscle spasms after SCI may be attributed to the neural circuits related to V3 SpINs. On the other hand, when they are silenced via an optogenetic approach, sensory-evoked spasms are inhibited at the same time (Lin et al., 2019). Taken together, these findings indicate that V3 SpINs contribute to the generation of sensory-mediated spasms after SCI.

Notably, these muscle spasms can be inhibited by repeated subthreshold activation of V3 SpINs (Lin et al., 2019). One possibility is that V3 SpINs may be overwhelmed so that they no longer respond to sensory input. Another possibility is that repeated stimulation may activate inhibitory interneuron-related neuronal pathways, which is consistent with the connection between V3 SpINs and other inhibitory interneurons (Lin et al., 2019). Consequently, suprathreshold stimulation of V3 SpINs leads to sensory-evoked muscle spasms, whereas subthreshold stimulation can only initiate coordinated locomotion patterns without the generation of muscle spasms (**Figure 4** and **Additional Table 1**).

In addition, the application of N-methyl-D-aspartate can reset and increase spasm activity, which leads to long EPSPs in motor neurons (Lin et al., 2019). Therefore, sensory inputs may activate N-methyl-D-aspartate receptors in V3 SpINs or other interneurons within the CPG circuitry after SCI, which leads to EPSPs in motor neurons and then muscle spasms. Notably, spasms do not occur in the uninjured spinal cord, and V3 SpINs only contribute to a coordinated locomotor pattern instead of initiating locomotion in the intact spinal cord (Zhang et al., 2008). In summary, V3 SpIN-related neural circuits experience neuroplasticity after SCI.

As noted above, V3 SpINs are part of the sensory PAD circuit, leading to either GABA-mediated or glutamate-mediated PAD. They also act as propriospinal neurons, projecting over many segments to expand the effect of the PAD. The intrinsic sodium-mediated persistent inward currents in V3 SpINs also allow this sensory transmission to last over a long period of time (Lin et al., 2023). After SCI, the glutamate-mediated PAD markedly increased, whereas the GABA-mediated PAD remained unchanged or decreased, suggesting that more innervation of V3 SpINs on sensory afferents increases and maintains the excitatory level after SCI (Lin et al., 2023), which seems to be related to the recovery of complex locomotion after SCI (**Figure 4** and **Additional Table 1**).

Overall, V3 SpINs help maintain excitation and facilitate sensory transmission to motor outputs after SCI, which is important for restoring residual motor functions.

## Therapeutic Potential of Spinal V3 Interneurons after Spinal Cord Injury

As highlighted here, V3 SpINs play diverse roles in controlling motor functions normally and contribute to neural circuit remodeling and the modulation of motor neuron excitability after SCI. Thus, they seem to be attractive therapeutic targets for SCI. However, no effective strategies targeting V3 SpINs have yet been developed in clinical practice. Consequently, we propose how to harness V3 SpINs for SCI on the basis of existing evidence.

### Harnessing host spinal V3 interneurons to repair the injured spinal cord

Therapeutic strategies targeting the neuroplastic potential of interneurons after SCI include activity-based therapies, neural interfacing, and cell therapies (Dominici et al., 2012; Rowald et al., 2022; Svendsen and Svendsen, 2024). Although the unique molecular characteristics of V3 SpINs have been identified (Deska-Gauthier et al., 2024), we do not yet have direct access to the key neural circuits related to these interneurons for activity-based therapies and neural interfacing. Therefore, harnessing host V3 SpINs to repair the injured spinal cord has become a challenge.

**Figure 6:**
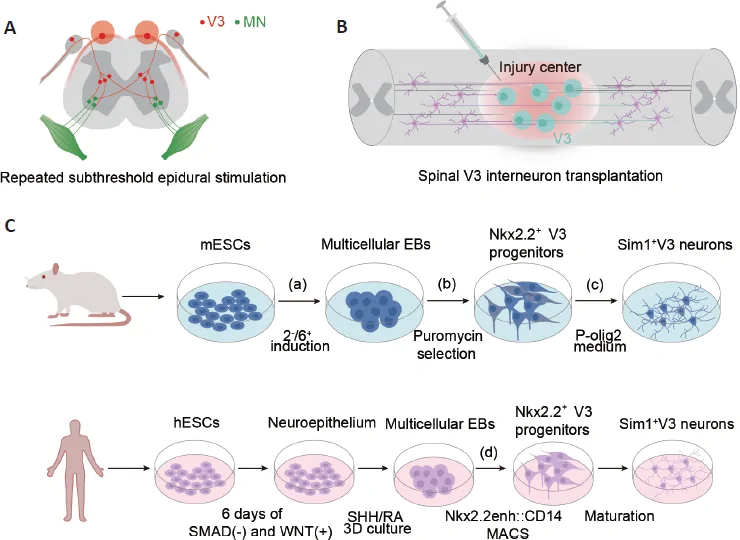
Prospective therapeutic strategies targeting spinal V3 interneurons after SCI. (A) Harnessing host V3 SpINs to repair the injured spinal cord. V3 SpINs receive abundant sensory inputs and connect ipsilateral and contralateral motor neurons. In addition, repeated subthreshold stimulation of V3 SpINs can initiate complex coordinated locomotion patterns. Therefore, repeated subthreshold epidural stimulation may provide indirect pathways to target V3 SpINs for treating SCI. (B) V3 SpINs are transplanted to establish neural circuits after SCI. Given that V3 SpINs connect with various host neurons from the spinal cord and supraspinal regions, the grafting of spinal V3 progenitor cells into the injured spinal cord can help reconstitute damaged neural circuits and promote functional recovery after SCI. (C) V3 SpINs can be generated from both mESCs and hESCs. For cells derived from mESCs, an 8-day induction protocol (a) was established. The first 2 days of cell aggregation do not include RA or the Shh agonist. After that, a lower concentration of RA and longer exposure to the Shh agonist are needed to increase the production of spinal V3 neural progenitor cells through the end of induction (6 days) (2^–^/6^+^ induction). Puromycin selection for the desired cell populations was subsequently applied (b). After selection, conditioned glial medium is used to improve the survival and maturation of V3 SpINs (c). For the generation and enrichment of hESC-derived V3 SpINs, a high concentration of Shh agonist and 3D culture of cells were used. To enhance the purification of V3 SpINs, the membrane marker CD14 derived from the spinal V3-specific gene Nkx2.2 is enriched in spinal V3 progenitor cells and later V3 SpINs (d). EBs: Embryoid body; hESCs: human embryonic stem cells; MACS: magnetic-activated cell sorting; mESCs: mouse embryonic stem cells; MN: motor neuron; RA: retinoic acid; SCI: spinal cord injury; Shh: Sonic hedgehog; SpINs: spinal interneurons.

Traumatic SCI interrupts spinal neural circuits above and below the injury site, thus disrupting the mutual communication between the brain and the spinal cord (Anderson et al., 2022). Therefore, task-specific sensory information becomes the main source of motor control to produce complex motor behaviors in spinal CPGs (Courtine et al., 2009; Akay et al., 2014; Takeoka et al., 2014; Takeoka and Arber, 2019). V3 SpINs receive abundant sensory inputs in the intact spinal cord, project across the midline and between spinal segments, and ultimately depolarize motor neurons and locomotor-related interneurons (Lin et al., 2023; Zhang et al., 2025). Therefore, the recruitment of V3 SpINs by afferent nerve stimulation or epidural stimulation may provide indirect access to these interneurons to facilitate sensory transmission and depolarize motor outputs (Lin et al., 2019), which may help maintain the sustainable excitability of damaged spinal networks and augment motor recovery after SCI (**Figure 6**). Additionally, a previous study demonstrated that strong suprathreshold activation of V3 SpINs evoked muscle spasms, whereas repeated subthreshold stimulation instead reduced spasms (Lin et al., 2019). Thus, repeated weak stimulation of V3 SpINs can not only reduce spasms but also increase general patterned activity after SCI to promote the recovery of residual motor functions (Xu et al., 2015; Xu and Sakiyama-Elbert, 2015; Lin et al., 2019; Berzanskyte et al., 2023; Laflamme et al., 2023; **Figure 6** and **Additional Table 3**). Neural interfacing therapy targeting V3 SpINs holds significant promise for improving neurological function following SCI. Given the complex neural connections between V3 SpINs and both sensory inputs and supraspinal neurons, electrical stimulation of V3 SpINs not only directly modulates the excitability of these neurons but also facilitates the engagement of residual, anatomically intact neural projections with spinal circuits. This ultimately leads to the activation of motor neurons and the restoration of activity in paralyzed muscles. However, the neuromodulation approaches based on the stimulation of V3 SpINs present at least three key considerations that warrant attention. First, a prerequisite for the application of neuromodulation therapy is that the injury must be incomplete in severity, thereby allowing residual intact neural circuits to reconstruct connections with V3 SpINs and integrate into functional spinal neural circuits. Second, the approaches based on stimulation of V3 SpINs are poorly specific. The unique molecular characteristics of these neurons remain poorly defined, thereby hindering the precise targeting of electrical currents to this specific neuronal subpopulation. Alternatively, V3 SpINs represent a population of excitatory interneurons whose excitability is preserved following SCI. Consequently, the excessive recruitment of these neurons into sensory networks may contribute to increased pain or spasticity (Zholudeva et al., 2018a). Therefore, stimulation parameters, including amplitude and frequency, should be precisely adjusted. In addition, neurointerfacing therapy can be integrated with activity-based therapies and pharmacological neuromodulation utilizing monoaminergic agents to synergistically enhance functional recovery (Courtine et al., 2009). Activity-based interventions, including exercise and rehabilitation, engage specific neuronal subpopulations within neural circuits through task-specific, activity-dependent neuroplasticity mechanisms (Streeter et al., 2017). Given that V3 SpINs are locomotor-related neurons and we have observed their activation during treadmill walking (Zhang et al., 2022), it is postulated that these neurons may be recruited and activated during locomotor training, potentially contributing to motor recovery following SCI. However, a limitation of activity-based therapies is that they also primarily depend on the activation and recruitment of preserved neural pathways to induce neuroplasticity. Consequently, the underlying mechanisms involved in leveraging host V3 SpINs to facilitate SCI repair are the stimulation and activation of these excitatory INs to establish sustainable excitability within damaged neural circuits and promote plasticity in preserved spinal networks to restore motor function.

**Additional Table 3:** Therapeutic advances in targeting V3 neurons for SCI

Host	System	Method	Functional response	Reference
Sacrocaudal spinal cord from Sim1// ChR2 mice	Sacral spinal cord transection	Optogenetical activation or sensory stimulation of V3 neurons	Complex coordinated pattern of motoneuron activity	Lin et al., 2019
Adult Sim1//ChR2 mice	Sacral spinal cord transection	Repeated subthreshold light trains/strong suprathreshold light trains	Reduced muscle spasms; evoked muscle spasms	Lin et al., 2019
Sacrocaudal spinal cord from Sim1// ChR2 mice	Sacral spinal cord transection	Optogenetical activation or sensory stimulation of V3 neurons	Increased glutamate-mediated PAD (suggesting a role in motor recovery)	Laflamme et al., 2023
mESC-derived V3 INs	*In vitro* enrichment	Lower concentration of RA and longer duration of Shh agonist exposure	About 8% of the induced cells are V3 INs	Xu and Sakiyama- Elbert, 2015
mESC-derived V3 INs	*In vitro* enrichment	Puromycin selection	About 83% of the induced cells are V3 INs; electrophysiology demonstrates their functional maturation	Xu et al., 2015
hESC-derived V3 INs	*In vitro* enrichment	Higher concentration of Shh agonist/in a 3D format/ transgenic surface marker for MACS	About 95% of the induced cells are V3 INs	Berzanskyte et al., 2023
Adult female nude rat	Thoracic SCI	hESC-derived V3 neurons transplantation	V3 neurons survived, migrated and differentiated into mature neurons. They functionally integrated into host neural circuits and help restore motor functions following SCI.	Data not published

hESCs: Human embryonic stem cells; IN: interneuron; MACS: magnetic-activated cell sorting; mESCs: mouse embryonic stem cells; RA: retinoic acid; SCI: spinal cord injury; Shh: Sonic hedgehog.

### Transplanting spinal V3 interneurons to repair the injured spinal cord

In addition to activity-based therapies and neural interfacing, cell therapies, such as cell transplantation, are also strategies for treating SCI and have already entered into early clinical trials (Zipser et al., 2022; Ribeiro et al., 2023; Svendsen and Svendsen, 2024; Kirkeby et al., 2025). In particular, the transplantation of neurons to reconstitute damaged neural circuits is promising and can promote functional recovery after SCI (Kumamaru et al., 2018; Fischer et al., 2020). Among the neuronal subtypes, SpINs are considered good candidates because of their potential plasticity (Zholudeva et al., 2018a, 2021). For example, the transplantation of excitatory V2a SpINs has been shown to contribute to anatomical phrenic plasticity after cervical SCI (Zholudeva et al., 2018b), whereas the transplantation of inhibitory SpINs attenuates pain and spasm-associated symptoms (Fandel et al., 2016; Gong et al., 2021; Xu et al., 2022; Zheng et al., 2023a).

Owing to their extensive spinal network integration and functional involvement within the uninjured spinal cord and their potential contribution to neuroplasticity after SCI, V3 SpINs can act as viable candidates for cell transplantation therapy, promoting locomotor recovery after SCI. However, the derivation of them requires available cell sources and efficient differentiation methods (**Figure 6** and **Additional Table 3**).

The use of pluripotent stem cells allows specific neuronal subtypes to be generated *in vitro* (White and Sakiyama-Elbert, 2019). Initially, the cell source for deriving V3 SpINs is mouse embryonic stem cells (Xu and Sakiyama-Elbert, 2015; Sternfeld et al., 2017; **Figure 6**). Generally, neurons from the ventral spinal cord can be generated from mouse embryonic stem cells by exposing embryoid bodies to Shh pathway agonists and retinoic acid. Increasing Shh signaling, prolonging the duration of Shh exposure, and decreasing the retinoic acid concentration ensure the production of V3 SpINs (Xu and Sakiyama-Elbert, 2015). However, the yield of V3 SpINs by this differentiation protocol is relatively low (8%). Besides, this differential protocol generated other heterogeneous populations without a stringent selection strategy, which makes cell identification and characterization difficult. More importantly, heterogeneous populations with low purity of V3 SpINs are not suitable for cell transplantation, which can generate teratomas due to the incomplete differentiation and maturation of transplanted cells. To overcome this limitation, another differentiation protocol attempted to reduce heterogeneity by fluorescence-activated cell sorting (FACS). The FACS method requires either a unique cell surface marker or genetic engineering of a fluorescent marker to isolate pure cell populations (Singh Roy et al., 2005). After a high concentration of SAG with retinoic acid was applied to differentiate the mouse embryonic stem cells into neurospheres, V3 SpINs generated with the Sim1 reporter Cre/tdTomato were dissociated and quantified via FACS. This protocol can lead to high purification (98%) of V3 SpINs (Sternfeld et al., 2017). However, FACS increases the risk for contaminated cultures and may lead to low viability for post-mitotic neurons. Consequently, Xu et al. (2015) used antibiotic resistance to generate desired cell lines. They inserted the puromycin resistance enzyme puromycin-N-acetyltransferase into the Sim1 locus, allowing the enrichment of V3 SpINs via puromycin selection. This cell selection protocol achieved high purity (83%) of Sim1^+^ V3 SpINs (**Figure 6**). However, the cell source of murine nature limits V3 SpINs to be harnessed in the clinical settings. Later, human pluripotent stem cells, are also tailored to create V3 SpINs, which will facilitate the study of human diseases (**Figure 6**). To improve the purity of p3 progenitor cells and subsequent postmitotic V3 interneurons, a transgenic surface marker for Nkx2.2^+^ p3 progenitor cells named human CD14 was designed, and these labeled cells were isolated via subsequent magnetic-activated cell sorting. Using CD14-based magnetic-activated cell sorting, p3 progenitor cells can be enriched to more than 90%, and later differentiating Sim1^+^ postmitotic V3 SpINs can also be enriched (Berzanskyte et al., 2023; **Figure 6**). Considering the importance of harnessing neuronal plasticity for the treatment of human spinal disorders, our research group has already differentiated human pluripotent stem cells into specific neuronal subpopulations and used them for cell transplantation. For example, we differentiated human pluripotent stem cells into mature motor neurons with high purity (more than 90%) (Chen et al., 2014, 2015; Du et al., 2015), and we also obtained a high purity of 90% dI4 interneurons (90% Ptf1a^+^) for transplantation after SCI (Gong et al., 2021; Xu et al., 2022; Zheng et al., 2023a, b). On the basis of the successful derivation strategy of spinal motor neurons and dI4 SpINs, our group adopted a plurality of groups of different small molecule compositions to perform multi-stage induced differentiation on human embryonic stem cells and human induced pluripotent stem cells and also differentiated V3 SpINs to a purity of 60% (60% Sim1^+^). We transplanted differentiated spinal V3 cells into the rat injured spinal cord and found that grafted cells survive long-term in the lesion epicenter and mature into spinal V3 neurons (data not published). Thus, the cell sources of human pluripotent stem cells provide a new opportunity for us to differentiate V3 SpINs for cell transplantation in future clinics. Biological strategies involving the transplantation of neurons derived from human pluripotent stem cells, particularly specific interneuron subpopulations, contribute to the reconstitution of damaged neural circuits and enhance functional recovery, especially in cases of anatomically complete SCI. However, the extent of recovery achieved to date has remained insufficient to warrant clinical translation. Differentiating high purity of neuronal subpopulation and guiding their regrowing axons to relevant targets may be necessary to achieve recovery (Squair et al., 2023). Moreover, the sole transplantation of neurons into the injured spinal cord may induce maladaptive plasticity within neural circuits, resulting in the deterioration of neurological function (Beauparlant et al., 2013). Therefore, complementary interventions, such as rehabilitative therapies, should be incorporated to improve therapeutic efficacy. The administration of cellular transplants in combination with regenerative compounds or biomaterials can further promote neurite outgrowth and facilitate the re-establishment of viable neural circuits. Overall, V3 SpINs can serve as important therapeutic targets for treating human spinal disorders, and it is clear that the combination of multiple therapeutic strategies may offer a greater potential for spinal cord repair (**Figure 6**).

## Limitations

We conclude here the functional roles of V3 SpINs in motor regulation through our review of related research and reviews and hold promise that V3 SpINs could be potential therapeutic targets for spinal cord disorders in future clinics. However, despite its promise, there also exist several limitations that deserve consideration. First, the specific mechanisms that underlie the motor regulation of V3 SpINs are obscure, such as the underlying neural circuit and molecular mechanisms. Emerging advanced technologies, including neuroimaging, optogenetics, and gene editing technologies, may help in-depth study. Additionally, current studies predominantly focus on their integrative roles in CPG networks, neglecting their sensory integrative roles. For instance, it is known that these neurons receive both proprioceptive and non-proprioceptive sensory inputs (Lin et al., 2019, 2023; Laflamme et al., 2023). However, limited information is currently available regarding the synaptic connections between various primary afferents, V3 SpINs, other interneurons, and projection neurons, which may uncover additional functional roles of V3 SpINs in sensory-motor transmission. Furthermore, the plasticity of sensory neural circuits following SCI not only contributes to alterations in motor function but also gives rise to abnormal pain sensations, such as hyperalgesia, allodynia, and spontaneous pain, as well as the potential manifestation of itch (Todd, 2010; Plant et al., 2018). However, the potential crosstalk between V3 SpINs and dorsal horn circuits has not yet been addressed, nor have their roles in contributing to sensory dysfunctions following SCI. Furthermore, V3 SpINs are highly heterogeneous, which contain various subtypes. The recent studies do not resolve which specific subpopulations are responsible for specific motor regulation. Finally, some spinal interneurons are not necessary for locomotion in the intact spinal cord, but are important for the motor recovery after SCI (Bui et al., 2016). The underlying mechanisms may be attributed to their identity transformation, such as V2a SpINs becoming more sensitive to serotonin after SCI (Husch et al., 2012; Garcia-Ramirez et al., 2021; Kathe et al., 2022). However, the functional roles of V3 SpINs in spinal cord disorders and the underlying mechanisms are not comprehensively defined in the promising preclinical research, which impedes the harnessing of their therapeutic potential in clinical practice. Therefore, although V3 SpINs show promise in treating diseases, their practical application still faces many limitations, which need to be resolved in future research.

## Conclusion and Future Perspectives

V3 SpINs are a highly heterogeneous group that are distributed along the dorsal-ventral and rostral-caudal axes in the mature spinal cord. While V3 SpINs generally function to ensure robust and balanced locomotion, different V3 subpopulations exhibit distinct functions in locomotion (Borowska et al., 2013). This is in accordance with different spinal V2a subtypes in adult zebrafish, which form unique neural circuits and contribute to distinct locomotor frequencies (Ampatzis et al., 2014). However, the functional roles of different spinal V3 subtypes are not yet fully understood. We now have atlases of the genetic expression of neuronal subtypes in both developing (Delile et al., 2019; Osseward et al., 2021) and adult (Sathyamurthy et al., 2018; Russ et al., 2021) mouse spinal cords, as well as in developing (Rayon et al., 2021; Zhang et al., 2021; Andersen et al., 2023; Li et al., 2023; Shi et al., 2024) and adult (Yadav et al., 2023; Zhang et al., 2024) human spinal cords. Using single-nucleus RNA-seq and spatial transcriptomics, diverse neuronal clusters with distinct spatial distributions were identified. Therefore, future work should focus on the identification of V3 SpINs and their subtypes in the human spinal cord, which is necessary for understanding their distinct roles in neuronal circuit formation and motor behavior regulation.

SpINs are distributed along the rostro-caudal axis of the spinal cord at the cervical, thoracic and lumbar levels (Francius et al., 2013). Despite their continuity along the spinal cord, they form distinct neural networks and control different motor behaviors at different spinal levels. For example, lumbar V2a SpINs have intense connections with the local CPG circuit, contributing to the left-right coordination of hindlimbs, whereas cervical V2a SpINs project to supraspinal regions and are essential for forelimb reaching (Crone et al., 2009; Azim et al., 2014; Pivetta et al., 2014; Hayashi et al., 2018). Previous studies have provided a deep understanding of the role of lumbar V3 interneurons in controlling hindlimb locomotion (Zhang et al., 2022). However, the anatomical locations, neural circuit connections, and functional roles of cervical V3 SpINs have not been fully explored, which may be related to skilled motor functions. Thus, future studies need to focus on the identification of cervical V3 SpINs.

A recent study generated a whole-brain map of monosynaptic inputs to V1 SpINs via viral transsynaptic tracing along with serial two-photon tomography (Chapman et al., 2025). Given the direct intraspinal connection between lumbar V3 interneurons and thoracic sympathetic networks, V3 SpINs may be involved in more complicated neural networks and play different functional roles other than simple motor control (Chacon et al., 2023). Moreover, sensory neural circuits, including primary afferents and their reciprocal interactions with dorsal horn neurons play a crucial role in transmitting sensory information to higher brain centers. In addition, these neural circuits transmit nociceptive signals to the ventral horn of the spinal cord and play a role in spinally mediated nocifensive reflexes (Todd, 2017; Hughes and Todd, 2020; Peirs et al., 2020). More importantly, the plasticity of sensory neural circuits following SCI may also lead to the development of neuropathic pain and itch (Todd, 2010; Plant et al., 2018). Given that V3 SpINs are glutamatergic and exhibit complex axonal projections, the potential crosstalk between V3 SpINs, primary afferents, and dorsal horn neurons is not only essential for modulating sensorimotor functions, but also implicated in the management of motor dysfunctions, neuropathic pain, and other neurological disorders under pathological conditions. The structural evidence obtained through neural circuit tracing and tissue-clearing technology, as well as functional evidence from dual optogenetics approaches combined with calcium imaging in future work, may help elucidate the complex connectivity patterns of V3 SpINs with supraspinal regions, intraspinal circuits, and sensory systems, thereby endowing them with multiple diverse functions.

SpINs not only play crucial roles in the regulation of sensory and motor functions within the uninjured spinal cord but also contribute to neuroplasticity following SCI, which can be harnessed to promote functional recovery and contribute to spinal cord repair (Zholudeva et al., 2018a, 2021). We know that V3 SpINs retain their excitability and facilitate sensory transmission to motor neurons after SCI. Their excessive recruitment into sensorimotor networks after SCI may both lead to spasticity and coordinated locomotion patterns (Lin et al., 2019). Therefore, whether we can therapeutically harness their positive effects and avoid maladaptive plasticity-related adverse effects is also an unresolved problem that needs to be addressed in the future.

Functional recovery following SCI predominantly relies on the reorganization of specific neuronal subpopulations. For instance, the recruitment of V2a SpINs through EES and cell transplantation promotes the recovery of walking and breathing, suggesting these cells interact with the related neural circuits that produce these functions (Zholudeva et al., 2017; Kathe et al., 2022). Likely, neurons located in spinal dorsal (dI4) and deep intermediate layers (dI3) have also been implicated (Bui et al., 2016; Gong et al., 2021). Consequently, these observations underscore the necessity of focusing on well-defined neuronal subpopulations. Single-cell technologies have revealed a remarkable diversity of neuronal subpopulations within the spinal cord, particularly based on recent single-cell RNA sequencing data from adult human spinal cord (Yadav et al., 2023; Zhang et al., 2024). These advancements are expected to facilitate the identification of recovery-organizing neurons following SCI and contribute to future clinical translation. Furthermore, functional recovery may also depend on the ability to differentiate NPCs into specific neuronal subpopulations. Extrinsic factor-mediated differentiation approaches, such as those involving membrane markers like CD14, which is regulated by specific transcription factors associated with particular neuronal subpopulations can facilitate the isolation and enrichment of these cell types (Berzanskyte et al., 2023). Similarly, the CRISPR-based lineage tracing techniques can be employed to pre-label specific neuronal subpopulations, enabling their subsequent tracking and isolation, thereby addressing the challenges associated with subtype-specific differentiation.

Given that the limited functional recovery observed thus far is insufficient for effective clinical translation, we propose that direct intervention targeting specific neuronal subpopulations may be necessary to enhance recovery. In addition to the reorganization of broadly defined neural circuits, targeted stimulation of specific neuronal subpopulations, such as through pharmacological agents (agonists/antagonists) or optogenetic approaches aimed at modulating specific neuronal signaling pathways may offer a more effective strategy. However, a prerequisite is to characterize the molecular dynamics underlying recovery from spinal cord disorders. Single-cell technology is essential for dissecting the molecular signatures and regenerative requirements of specific neuronal populations. Subsequently, each recovery-organizing neuronal subpopulation, such as V3 SpINs, can be harnessed to activate and reconstruct their projections to corresponding target regions. In conclusion, there is a growing effort to identify V3 SpINs and their contributions to motor, sensory, and autonomic functions. With the advantages of genetic and virus tracing technology, multiple researchers are providing a better understanding of this specific interneuron population. However, more detailed information on the identity and function of V3 SpINs, as well as their therapeutic effects after SCI, has not been elucidated. Thus, ongoing work needs to characterize them and their subtypes and improve our understanding of their contribution to the recovery of functions after SCI. In this way, effective treatments can be developed to selectively target their therapeutic potential.

## Additional files:

***Additional Table 1:*** *Roles of spinal V3 interneurons in neural circuits and motor function.*

***Additional Table 2:*** *Functional roles of spinal V3 interneuron subpopulation.*

***Additional Table 3:*** *Therapeutic advances in targeting V3 neurons for SCI.*

## Data Availability

*All relevant data are within the paper and its Additional files.*
